# LncRNA NNT-AS1 contributes to the cisplatin resistance of cervical cancer through NNT-AS1/miR-186/HMGB1 axis

**DOI:** 10.1186/s12935-020-01278-9

**Published:** 2020-05-24

**Authors:** Yanjie Liu, Ruixia Guo, Yuhuan Qiao, Liping Han, Mingzhu Liu

**Affiliations:** 1 Department of Gynaecology, The First Affiliated Hospital of Zhengzhou University, No. 1, Jianshe East Road, Zhengzhou, 450052 China; 2 Gynaecologic Department of Traditional Chinese Medicine, The First Affiliated Hospital of Zhengzhou University, Zhengzhou, 450052 China

**Keywords:** NNT-AS1, miR-186, HMGB1, Cisplatin resistance, Cervical cancer

## Abstract

**Background:**

Cisplatin (DDP) is a major chemotherapeutic drug which was widely used for cervical cancer (CC) patients with advanced or recurrent although its limitation in the development of resistance. LncRNA nicotinamide nucleotide transhydrogenase-antisense RNA1 (NNT-AS1) has been reported to be involved in the DDP resistance. However, the role of NNT-AS1 in DDP resistance in CC remain unknown.

**Methods:**

The mRNA expression of NNT-AS1, microRNA-186 (miR-186) and HMGB1 was detected by quantitative real-time polymerase chain reaction (qRT-PCR). Cell proliferation and apoptosis abilities were measured via MTT assay or flow cytometry, respectively. Western blot was used to measure the expression level of HMGB1, Bax, Bcl-2, Cleaved-caspase 3, N-cadherin, Vimentin and E-cadherin. Cell migration and invasion abilities were analyzed using Transwell assay. The interaction among NNT-AS1, miR-186 and HMGB1 was confirmed by luciferase reporter assay and RNA pull-down assay. Murine xenograft model was established using stably transfected SiHa/DDP cells.

**Results:**

NNT-AS1 level was significantly elevated in CC tissues and cells, especially in DDP-resistant tumors and cell lines. Subsequently, loss-of function assays indicated that NNT-AS1 silence could attenuate DDP resistance by inhibiting proliferation, metastasis and EMT but inducing apoptosis in DDP-resistant CC cells. Besides that, knockdown of NNT-AS1 also antagonized DDP resistance in vivo. Bioinformatics predication revealed NNT-AS1 directly bound to miR-186 and HMGB1 was a target of miR-186. Additionally, NNT-AS1 could regulate HMGB1 expression via targeting miR-186. Furthermore, restoration experiments showed NNT-AS1 knockdown might improve DDP-sensitivity of CC cells via blocking HMGB1 expression by competitive interaction with miR-186.

**Conclusion:**

NNT-AS1 improved chemoresistance of DDP-resistant CC cells via modulating miR-186/HMGB1 axis.

## Background

Cervical cancer (CC) is the second most common female cancer worldwide, and is the leading cause of mortality from cancer among females in developing countries [1]. Though great advances aimed at the development of therapy for preventing CC in recent decades, about 20–25% patients remain suffer from treatment failure because of distant metastasis and recurrence [2, 3]. Cisplatin (DDP) is a widely used chemotherapeutic drug, and is often used to combination with radiotherapy for the treatment of patients with advanced or recurrent CC [4]. However, the development of resistance to DDP-based chemotherapy has become a major limiting factor for its efficacy as an anticancer drug [5].

Long non-coding RNAs (lncRNAs) are RNA transcripts longer than 200 nucleotides in length and are not translated into protein [6]. Nevertheless, emerging evidence has revealed that lncRNAs involve in multiple biological activities, such as gene regulation, RNA transport and protein synthesis [7, 8]. Recently, lncRNAs were evidently discovered to modulate drug transport and ultimate resistance [9]. Many lncRNAs are reported to mediate DDP resistance in cancers. For example, lncRNA XIST promoted DDP resistance of human lung adenocarcinoma cells via let-7i/BAG-1 axis [10]. LncRNA UCA1 promoted proliferation and DDP resistance of oral squamous cell carcinoma by suppressing miR-184 expression [11]. LncRNA CCAT1/miR-130a-3p axis increased DDP resistance in non-small-cell lung cancer cell line by targeting SOX4 [12]. In recent, LncRNA nicotinamide nucleotide transhydrogenase-antisense RNA1 (NNT-AS1), located in the chromosome 5p12 region, has been reported to be highly expressed in drug-resistant NSCLC tissues and cells, and promote the resistance of NSCLC cells to DDP through the MAPK/Slug signaling pathway [13], indicating NNT-AS1 may be related to DDP-resistant. Additionally, NNT-AS1 was verified to function as a tumor promoter in CC via promoting cell proliferation and invasion [14]. However, the relationship between NNT-AS1 and DDP resistance in CC has not been reported.

MicroRNAs (miRNAs), a class of small noncoding RNAs with 22 nucleotides in size, are able to bind to the 3′-untranslated region (3′UTR) of most protein-coding transcripts to lead translational repression, mRNA decay and mRNA deadenylation [15, 16]. Recently, increasing evidence has revealed that aberrantly expressed miRNAs involve in the development of drug resistance [17, 18]. MiR-186, a member of miRNAs, has also been found that can induce sensitivity of cancer cells to DDP in several tumors, including ovarian cancer, glioblastoma and lung cancer [19–21]. All the findings indicated the regulatory role of miR-186 in DDP resistance. High mobility group box 1 (HMGB1) protein is a ubiquitous chromatin component expressed in nucleated mammalian cells. HMGB1 has been identified to be involved in transcription regulation of many cancer genes, including BRCA1, E-selectin, TNF-α and insulin receptor [22]. Interestingly, recent evidence demonstrated that HMGB1 was overexpressed in CC and promoted cell invasion and migration in vitro [23]. Besides that, highly expressed HMGB1 contributed to the DDP resistance in human CC cells [24]. Nevertheless, the mechanisms underlying HMGB1 affects DDP resistance in CC remains unclear.

In the present study, we explored the expression patterns of NNT-AS1 in CC DDP-resistant tissues and cell lines and investigated the effects of NNT-AS1 on CC patients DDP resistance in vivo and *vitro.* Besides that, we also explored the potential molecular mechanisms underlying the function of NNT-AS1 on DDP resistance. This study may contribute to provide a potential therapeutic approach for CC treatment.

## Materials and methods

### Patients and specimens

The study was approved by the Ethics Committee of the First Affiliated Hospital of Zhengzhou University and written informed consents were collected from all patients and hospitals. Cervical cancer tissues and adjacent normal tissues were collected from 58 CC patients undergoing surgical resection in the First Affiliated Hospital of Zhengzhou University and all cancer tissue samples were diagnosed as CC by pathological examination. All fresh samples were snap-frozen and preserved in liquid nitrogen until further experiments. 58 CC patients were classified into two groups depending on the sensitivity of CC patients to chemotherapy drugs: chemotherapy-sensitive group (tumor remission after 6 cycles of chemotherapy, Chemosensitive group, N = 24) and chemotherapy-resistant group (tumor stabilization or progression after 6 cycles of chemotherapy, Chemoresistant group, N = 34). Additionally, 58 patients were divided into two groups based on the expression of NNT-AS1 to calculate the overall survival of all participants at the different periods (0, 20, 40, 60 month) after cisplatin treatment.

### Cell culture and transfection

Cervical cancer cell lines HeLa and SiHa were purchased from the American Type Culture Collection (ATCC, Manassas, VA, USA). The normal cervical epithelial cell line HaCaT was obtained from institute of Biochemistry and Cell Biology (Shanghai, China). HeLa and SiHa cells were cultured in increasing concentrations of cisplatin (Sigma, St. Louis, MO, USA) for over 6 months to establish cisplatin-resistant cell lines, HeLa/DDP and SiHa/DDP. All cells were maintained in Dulbecco’s modified Eagle’s medium (DMEM, Gibco, Carlsbad, CA, USA) supplemented with 10% fetal bovine serum (Gibco), 100 U/mL penicillin and 100 U/mL streptomycin (SigmaAldrich, Shanghai, China) at 37 °C with 5% CO_2_ in a humidified atmosphere.

The short hairpin RNA (shRNA) targeting NNT-AS1 (sh-NNT-AS1) and shRNA scramble control (sh-NC), pcDNA and pcDNA-NNT-AS1 overexpression vector (NNT-AS1), pcDNA-HMGB1 overexpression vector (HMGB1) were synthesized by Genepharma (Shanghai, China). The miR-186 mimic (miR-186), mimic negative control (miR-NC), miR-186 inhibitor (anti-miR-186) and inhibitor negative control (anti-NC) were purchased from RIBOBIO (Guangzhou, China). The transfection of miRNA mimics (10 nM) or vectors was performed using Lipofectamine™ 2000 reagent (Invitrogen, Carlsbad, CA, USA), when the HeLa/DDP and SiHa/DDP cells reached 50–60% confluence. Then cells were harvested for 48 h for the subsequent analysis.

### Quantitative real-time polymerase chain reaction (qRT-PCR)

Total RNA was extracted from CC cells and tissues using TRIzol reagents (Invitrogen). RNA was reversely transcribed into complementary DNA (cDNA) with the help of AMV reverse transcription kits (Takara, Dalian, China). Fluorescence qRT-PCR was performed using an SYBR Premix Ex Taq II kit (Takara) according to the manufacturer’s introduction. GAPDH or U6 was as internal control and the fold change was assessed using the 2^−ΔΔCt^ method. The specific primer sequences were listed as follows: NNT-AS1, forward, 5′-ACGTGCAGACAACATCTACCT-3′, reverse, 5′-TACAACACCTTCCCGCAT-3′; miR-186, forward, 5′-CGCGGATCCGGTTTACAGAACACCCATCAT-3′, reverse 5′-CCGCTCGAGGTGTTGACATTCACATGCTTC-3′; HMGB1, forward: 5′-GGAGAGATGTGGAATA-3′, reverse, 5′-GGGAGTGAGTTGTGTA-3′; U6, forward 5′-CTCGCTTCGGCAGCACA-3′, reverse 5′-ACGCTTCACGAATTTGCGT-3′; GAPDH, forward 5′-AACGGATTTGGTCGTATTGG-3′, reverse 5′-TTGATTTTGGAGGGATCTCG-3′.

### Cell viability assay

Cell viability was determined using the 3-(4,5)-dimethylthiahiazo (−z-y1)-3,5-di-phenytetrazoliumromide (MTT, Beyotime, Shanghai, China) assay. Briefly, transfected DDP-resistant cells were seeded into 96-well plate with a density of 5 × 10^3^ cells/well and incubated with different doses of DDP. At different time points, 20 μL of MTT solution was added to each well for 4 h, followed by the addition of DMSO to resolve the generated formazan. Finally, the absorbance was measured at 490 nm using a microplate reader (Bio-Rad, Hercules, CA, USA).

### Cell migration and invasion assay

The migration and invasive capacities of the DDP-resistant cells in vitro were detected by transwell assay. Transfected DDP-resistant cells were seeded in the upper chamber of a 24-well plate with or without matrigel (Becton–Dickinson, Franklin Lakes, NJ, USA) in DMEM without serum and complete medium with containing 10% FBS was added into the lower compartment of each well as a chemoattractant. After incubation for 24 h at 37 °C, the non-motile cells on the upper surface were removed using a dry cotton swab. Then cells attached to the bottom were fixed with methanol and stained with 0.5% crystal violet for 30 min. The cells from five randomly selected fields were counted with an inverted microscope.

### Cell apoptosis analysis

Transfected DDP-resistant cells were harvested for 48 h. Then Cell apoptosis rate was analyzed using Annexin V-FITC/PI apoptosis detection kit (Solarbio, Beijing, China) according to the instructions of manufacture. All samples were performed in triplicate.

### Western blot assay

Western blot assay was conducted as previous described [12]. Immunoblot assays were performed using antibodies against HMGB1, Bax, Bcl-2, Cleaved caspase 3, N-cadherin, Vimentin, E-cadherin, as well as GAPDH.

### Luciferase reporter assay

The NNT-AS1 mRNA and HMGB1 3′-UTR containing wild-type (WT) or mutant (MUT) binding sequence of miR-186 were cloned into the psiCHECK™-2 luciferase plasmid (Promega, Shanghai, China), respectively. Then, HeLa/DDP and SiHa/DDP cells were seeded in 24-well plates, followed co-transfected with NNT-AS1-WT or NNT-AS1-MUT or HMGB1-WT or HMGB1-MUT with miR-186 mimics or miR-NC respectively using Lipofectamine 2000 (Invitrogen). After transfection for 48 h, a dual luciferase assay kit (Promega) was used to analyze the luciferase activity following the manufacturer’s instructions.

### RNA pull-down assay

For the RNA pull-down assay, 3vitro-biotinylated miRNA mimic was transfected into DDP-resistant cells. Subsequently, cells were lysed and cultured with streptavidin-coupled beads to generate biotin-miRNA-lncRNA complexes. Finally. RNA was isolated and analyzed using qRT-PCR.

### Murine xenograft assay

Female BALB/c mice (aged 4–6 weeks) were bought from Vital River Laboratory Animal Technology (Beijing, China). The experiment was permitted by the Animal Research Committee of the First Affiliated Hospital of Zhengzhou University and performed in accordance with the guidelines of the National Animal Care and Ethics Institution. SiHa/DDP cells (5 × 10^6^) transfected with the sh-NNT-AS1 or sh-NC were subcutaneously injected into the backs of nude mice under sterile conditions. After 7-day administration, mice were treated with DDP or PBS followed by the examination of tumor sizes every 3 d. After 30 d, all mice were sacrificed and tumor samples were weighted and used for further molecular analysis.

### Statistical analysis

All statistical analyses were performed with GraphPad Prism 7 (GraphPad Inc., San Diego, CA, USA). The data were expressed as the mean ± SD. The correlation among NNT-AS1, miR-186 and HMGB1 was analyzed by Pearson’s correlation analysis. Kaplan–Meier survival curves were plotted and the difference in survival between two groups was analyzed by the log-rank test. The significant group differences were assessed by Student’s *t* test or one-way analysis of variance (ANOVA). *P *< 0.05 was considered statistically significant.

## Results

### NNT-AS1 is up-regulated in DDP-resistant CC tissues and cells lines and highly expressed NNT-AS1 predicates poor prognosis

**Fig. 1:**
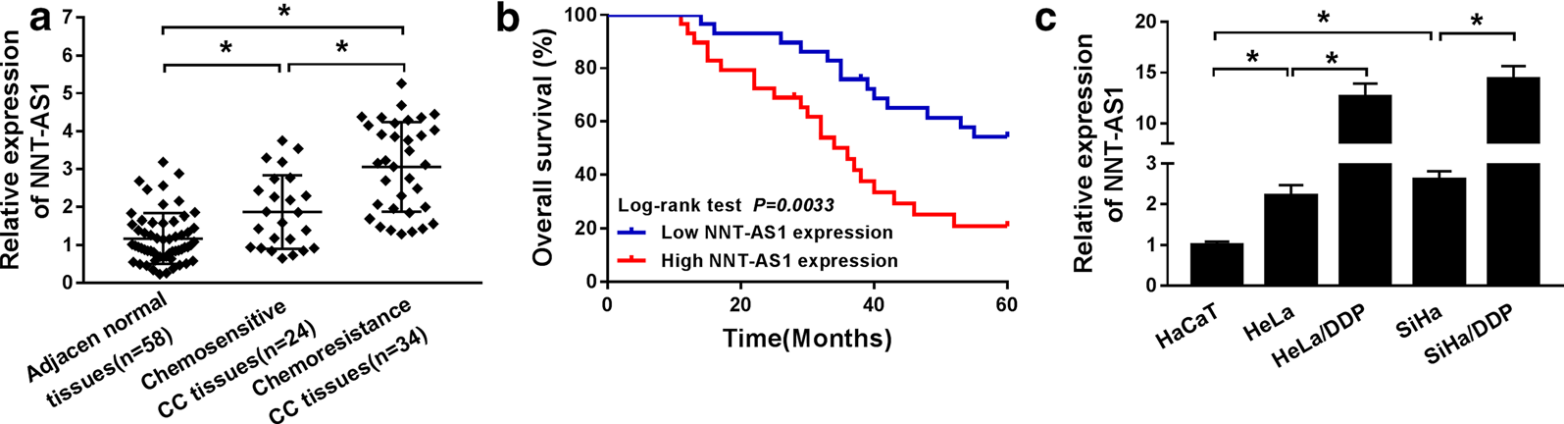
NNT-AS1 is up-regulated in DDP-resistant CC tissues and cells lines and highly expressed NNT-AS1 predicates poor prognosis. **a** NNT-AS1 expression was detected using qRT-PCR in normal tissues and DDP-sensitive or DDP-resistant CC tissues. **b** The role of NNT-AS1 in the CC prognosis was analyzed by Kaplan–Meier method. **c** NNT-AS1 level in HaCaT cells and DDP-sensitive or DDP-resistant cell lines was analyzed by qRT-PCR. *P < 0.05

To explore the role of NNT-AS1 in DDP-resistant CC, the expression of NNT-AS1 was detected using qRT-PCR in DDP-sensitive and DDP-resistant CC tissues and cells. Results showed that NNT-AS1 level was significantly elevated in CC tissues and cells, especially in DDP-resistant tumors and cell lines (HeLa/DDP and SiHa/DDP) (Fig. 1a, c). Additionally, 58 patients who underwent DDP treatment were followed up to 60-mouth for the analysis of prognosis and the results indicated that patients with higher level of NNT-AS1 expression had a poor overall survival compared to those with lower level of NNT-AS1 expression (Fig. 1b), indicating high NNT-AS1 expression predicated poor prognosis in CC patients.

### Knockdown of NNT-AS1 inhibits DDP-resistant CC cells viability and proliferation but induces apoptosis

**Fig. 2:**
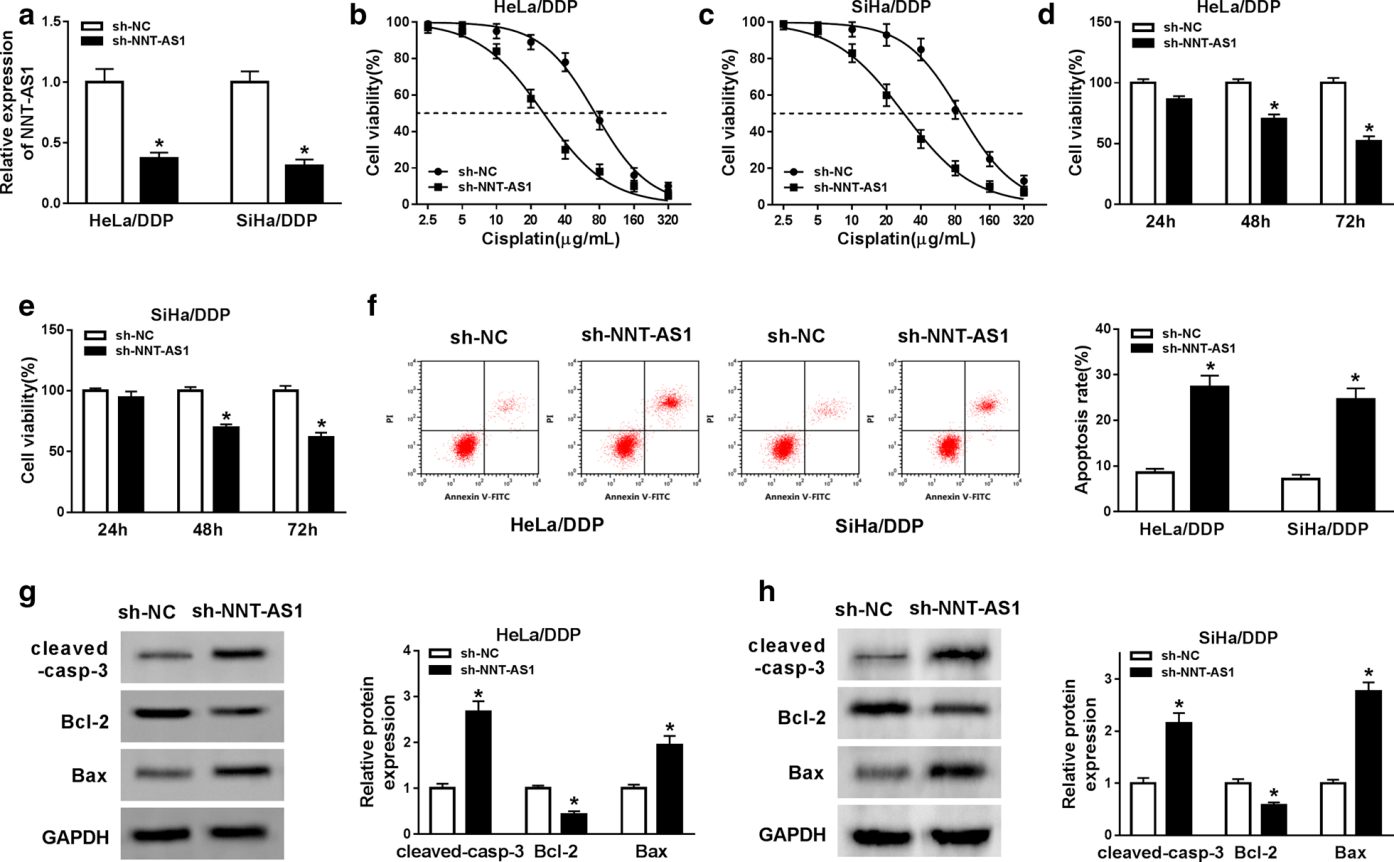
Knockdown of NNT-AS1 inhibits DDP-resistant CC cells viability and proliferation but induces apoptosis. **a** The expression of NNT-AS1 was measured in HeLa/DDP and SiHa/DDP cells after transfection with sh-NNT-AS1. **b**–**e** Cell viability and proliferation of HeLa/DDP and SiHa/DDP cells were detected by MTT assay. **f** The apoptosis rates were analyzed by Flow Cytometry in HeLa/DDP and SiHa/DDP cells. **g**, **h** The protein expression of cleaved caspase-3, Bcl-2 and Bax in HeLa/DDP and SiHa/DDP cells was detected by western blot. *P < 0.05

To further investigate the biological effects of NNT-AS1 on DDP resistance CC cells, HeLa/DDP and SiHa/DDP cells were transfected with sh-NC or sh-NNT-AS1. Then, the transfection efficacy was verified by qRT-PCR. The result showed that NNT-AS1 expression was decreased in cells transfected with sh-NNT-AS1 (Fig. 2a). After treatment with different concentrations of cisplatin for 48 h, we found the viability of HeLa/DDP and SiHa/DDP cells was decreased with the increase of DDP concentration, while NNT-AS1 deletion remarkably inhibited the proliferation ability of DDP resistant cells (Fig. 2b–e). In addition, the results of flow cytometry indicated that knockdown of NNT-AS1 significantly induced apoptosis of resistant cells (Fig. 2f). Meanwhile, western blot analysis results showed NNT-AS1 deletion promoted the expression of cleaved-caspase-3 and Bax but inhibited the level of Bcl-2 in HeLa/DDP and SiHa/DDP cells, further suggesting that knockdown of NNT-AS1 could enhance the apoptosis of resistant cells (Fig. 2g, h). In a word, knockdown of NNT-AS1 attenuated DDP resistance by inhibiting proliferation and inducing apoptosis in DDP-resistant CC cells.

### Knockdown of NNT-AS1 inhibits migration, invasion and EMT of DDP-resistant CC cells

**Fig. 3:**
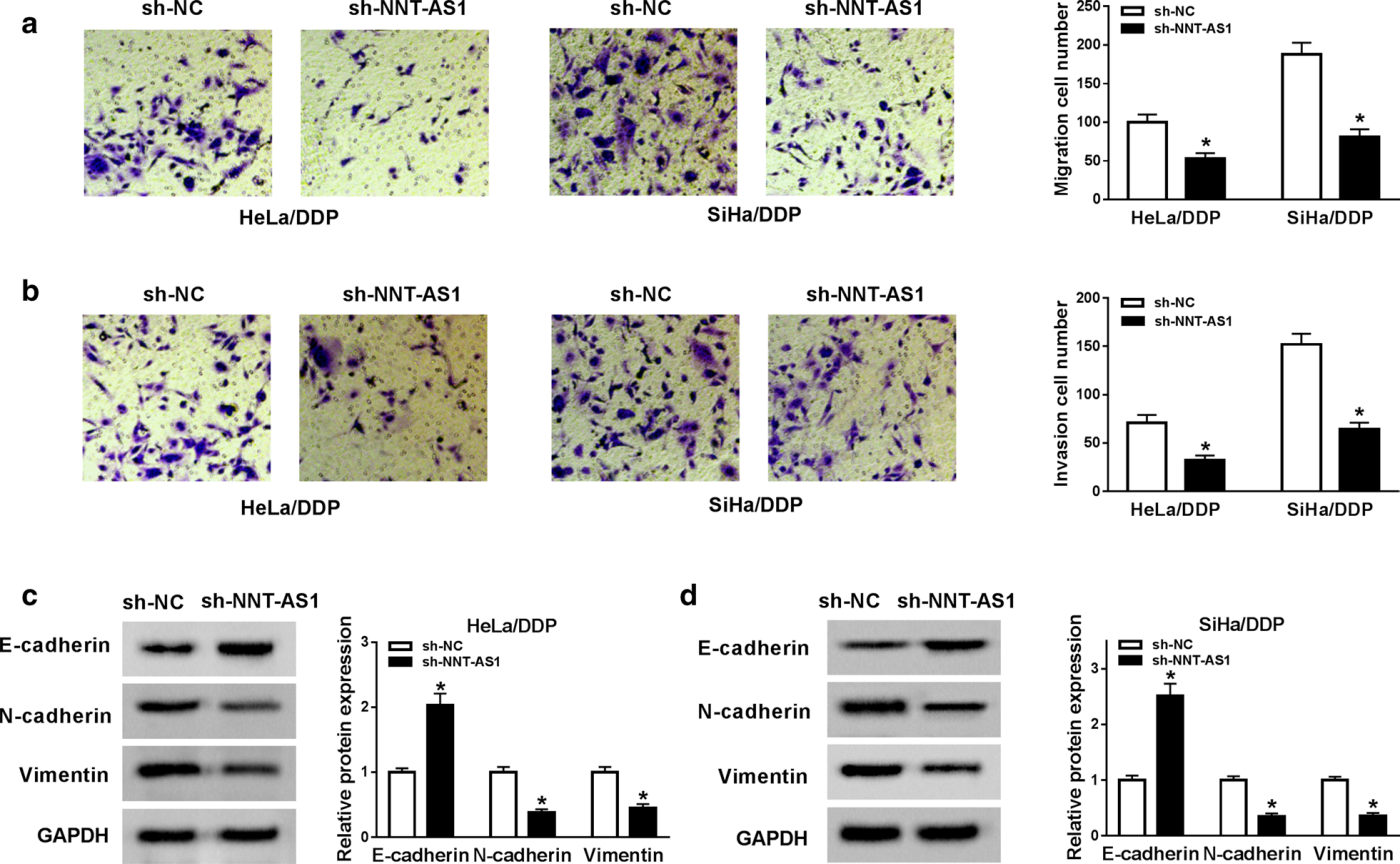
Knockdown of NNT-AS1 inhibits migration, invasion and EMT of DDP-resistant CC cells. **a**, **b** Transwell assay was used to analyze migration and invasion abilities of HeLa/DDP and SiHa/DDP cells. **c**, **d** The levels of E-cadherin N-cadherin and Vimentin were detected by western blot. *P < 0.05

Immediately, transwell assay was performed and the results showed knockdown of NNT-AS1 could suppress migration and invasion abilities of HeLa/DDP and SiHa/DDP cells (Fig. 3a, b). Furthermore, epithelial-mesenchymal transition (EMT) related protein was analyzed using western blot and an inhibition of N-cadherin and Vimentin expression but an enhancement of E-cadherin expression induced by NNT-AS1 deletion was investigated in HeLa/DDP and SiHa/DDP cells (Fig. 3c, d) indicating knockdown of NNT-AS1 could inhibit resistant cells EMT in CC. In all, we illustrated that knockdown of NNT-AS1 could attenuate DDP resistance by inhibiting proliferation metastasis and EMT in DDP-resistant CC cells.

### NNT-AS1 directly bind to miR-186 and negatively regulates its expression

**Fig. 4:**
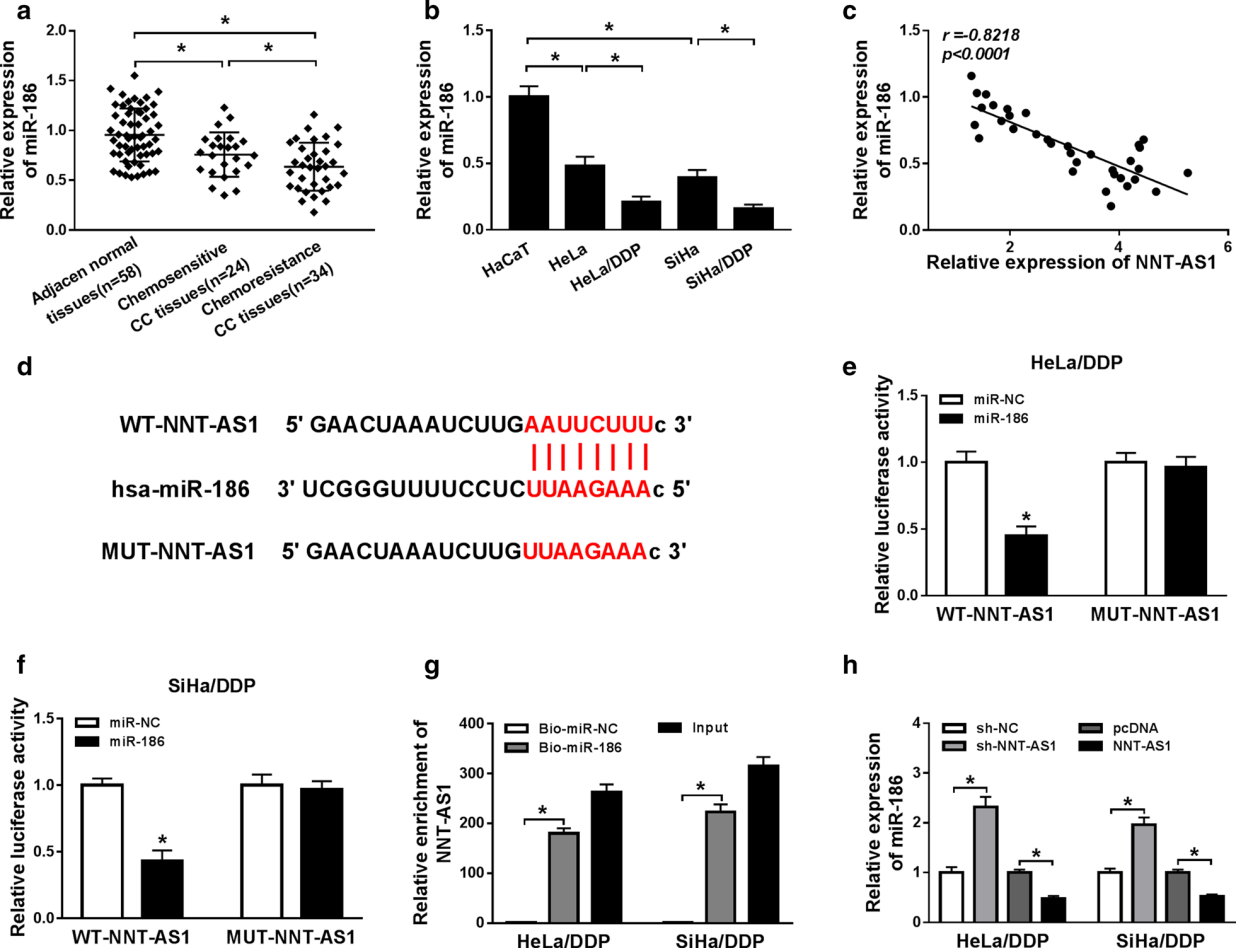
NNT-AS1 directly binds to miR-186 and negatively regulates its expression. **a**, **b** The expression of miR-186 in DDP-sensitive or DDP-resistant CC tissues and cell lines was detected using qRT-PCR. **c** The correlation between NNT-AS1 and miR-186 was analyzed. **d** The predicted binding sequences between NNT-AS1 and miR-186 were shown. **e**, **f** Luciferase reporter assays in HeLa/DDP and SiHa/DDP cells after the co-transfected with NNT-AS1-WT or NNT-AS1-MUT and miR-186 mimic or miR-NC were conducted. **g** The level of NNT-AS1 was measured using qRT-PCR in samples pulled down by biotinylated miR-186 or negative control. **h** QRT-PCR was used to determine the expression of miR-186 in HeLa/DDP and SiHa/DDP cells transfected with sh-NC or sh-NNT-AS1 or pcDNA or NNT-AS1. *P < 0.05

The expression of miR-186 was detected and we found miR-186 was down-regulated in CC tissues and cell lines compared to the normal tissues and cells, especially decreased in DDP-resistant tumors and cell lines (Fig. 4a, b). Subsequently, a perfect negative correlation between miR-186 and NNT-AS1 expression in CC patients was identified (R = − 0.8218, *P *< 0.0001) (Fig. 4c). All the results indicated that miR-186 might relate to NNT-AS1 medicated DDP-resistant. To elucidate this hypothesis, bioinformatics analysis was performed and miR-186 was predicted to be a target of NNT-AS1 with putative binding sites (Fig. 4d). Then luciferase reporter assay results showed miR-186 mimic transfection reduced the luciferase activities of the WT-NNT-AS1 reporter vector but not empty vector or MUT-NNT-AS1 reporter vector in HeLa/DDP and SiHa/DDP cells (Fig. 4e, f). In the meanwhile, The RNA pull-down assays further confirmed the direct interaction between miR-186 and NNT-AS1 because a significant enrichment of NNT-AS1 was measured in both HeLa/DDP and SiHa/DDP cells (Fig. 4g). Furthermore, qRT-PCR was carried out and we found NNT-AS1 inhibited miR-186 expression, inversely, NNT-AS1 deletion promoted miR-186 expression in HeLa/DDP and SiHa/DDP cells (Fig. 4h). Thus, NNT-AS1 was a sponge of miR-186 and negatively regulated its expression.

### Knockdown of NNT-AS1 attenuates DDP resistance in DDP-resistant CC cells through regulating miR-186

**Fig. 5:**
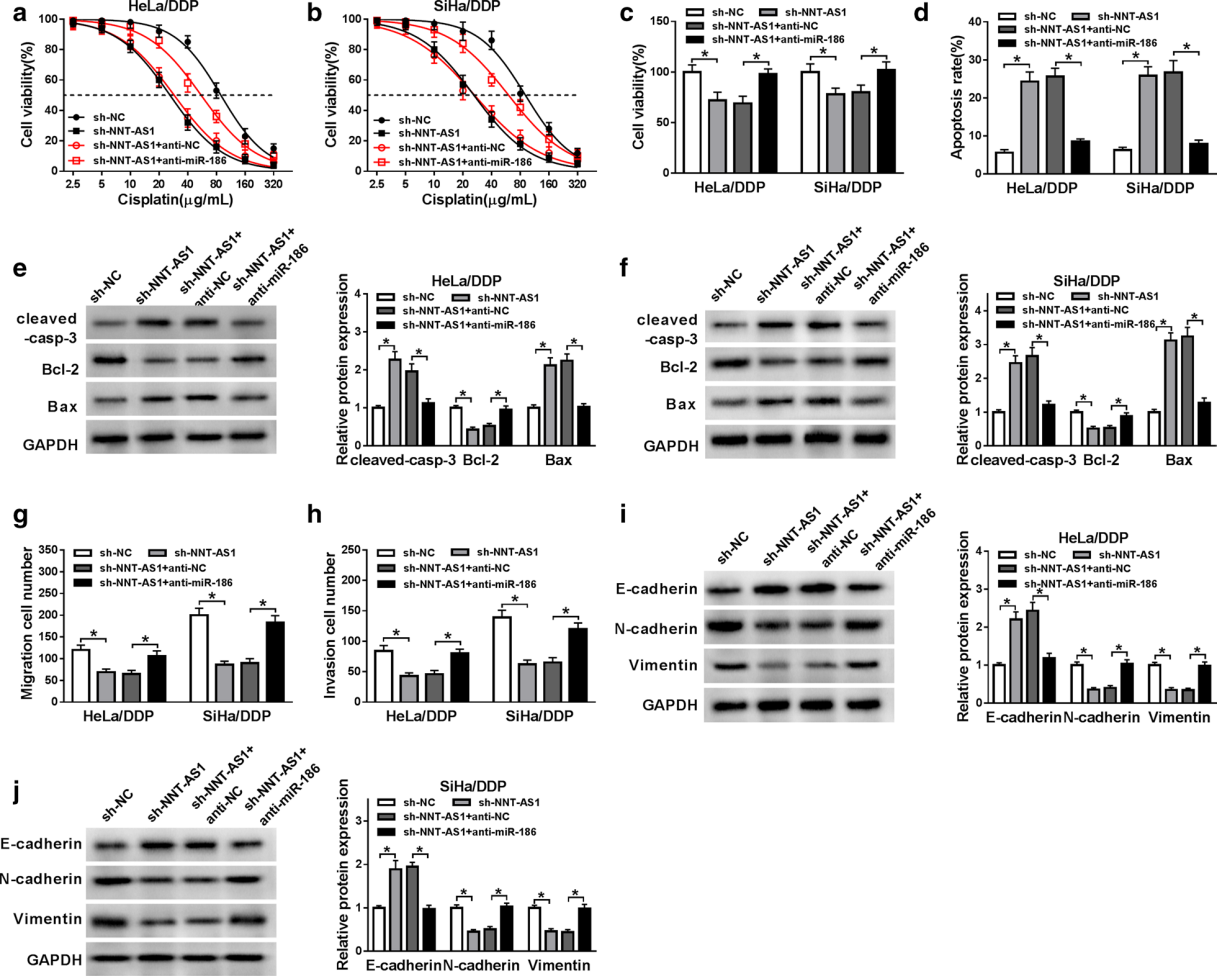
Knockdown of NNT-AS1 attenuates DDP resistance in DDP-resistant CC cells through regulating miR-186. HeLa/DDP and SiHa/DDP cells were transfected with sh-NC, sh-NNT-AS1, sh-NNT-AS1 + anti-miR-NC or sh-NNT-AS1 + anti-miR-186. **a**–**c** Cell viability and proliferation of HeLa/DDP and SiHa/DDP cells were detected by MTT assay. **d** The apoptosis rates were analyzed by Flow Cytometry in HeLa/DDP and SiHa/DDP cells. **e**, **f** The protein expression of cleaved caspase-3, Bcl-2 and Bax in HeLa/DDP and SiHa/DDP cells was detected by western blot. **g**, **h** Transwell assay was used to analyze migration and invasion abilities of HeLa/DDP and SiHa/DDP cells. **i**, **j** The levels of E-cadherin N-cadherin and Vimentin were detected by western blot. *P < 0.05

To explore whether miR-186 involved in the NNT-AS1 medicated DDP resistance, HeLa/DDP and SiHa/DDP cells were transfected with sh-NC, sh- NNT-AS1, sh-NNT-AS1 + anti-miR-NC or sh-NNT-AS1 + anti-miR-186. After transfection, the proliferation, apoptosis, migration, invasion and EMT abilities of DDP-resistant cells were investigated through restoration experiments. Interestingly, results showed that miR-186 inhibitor transfection could attenuate NNT-AS1 deletion-mediated inhibition of proliferation, migration, invasion and EMT abilities as well as the promotion of apoptosis abilities in HeLa/DDP and SiHa/DDP cells (Fig. 5a–j). Taking together, we known that knockdown of NNT-AS1 could attenuate DDP resistance in DDP-resistant CC cells by targeting miR-186.

### HMGB1 is a target of miR-186 and NNT-AS1 can regulate HMGB1 expression via targeting miR-186

**Fig. 6:**
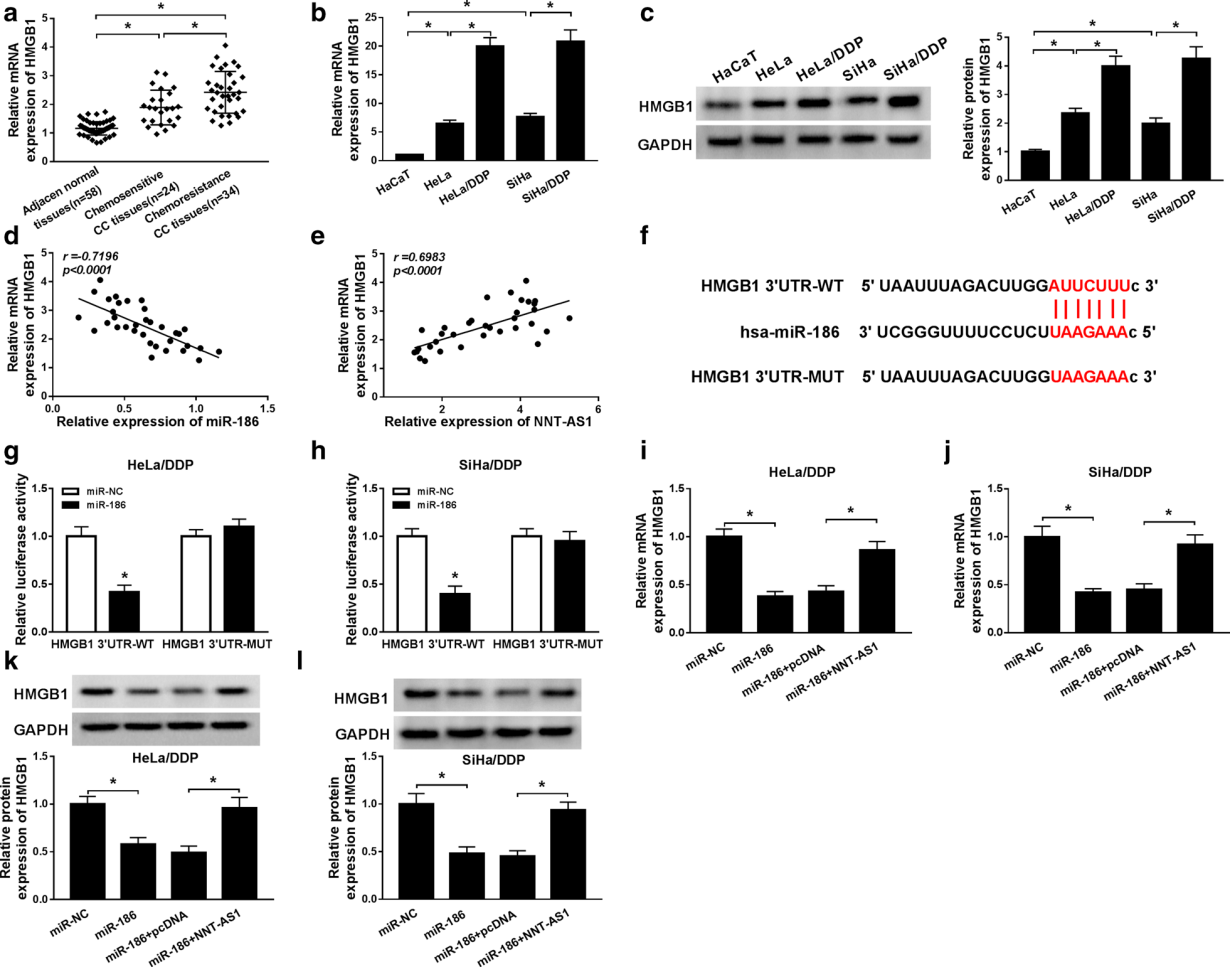
HMGB1 is a target of miR-186 and NNT-AS1 can regulate HMGB1 expression via targeting miR-186. **a**–**c** The expression of HMGB1 in DDP-sensitive or DDP-resistance CC tissues and cell lines was analyzed by qRT-PCR or western blot, respectively. **d** The correlation between HMGB1and miR-186 was determined. **e** The correlation between HMGB1and NNT-AS1 was analyzed. **f** The predicted binding sequences between HMGB1 and miR-186 were exhibited. **g**, **h** Luciferase reporter assay was conducted to evaluate the luciferase activity in HeLa/DDP and SiHa/DDP cells after the co-transfected with HMGB1-WT or HMGB1-MUT and miR-186 mimic or miR-NC. **i**–**l** QRT-PCR or western blot was used to analyze the expression of HMGB1 in HeLa/DDP and SiHa/DDP cells transfected with miR-NC or miR-186 or miR-186 and pcDNA or miR-186 and NNT-AS1. *P < 0.05

The mRNA expression of HMGB1 was found to be elevated in both CC tissues and cells line compared with the normal tissues and cells, especially in DDP-resistant CC tissues and cells lines (Fig. 6a, b). Besides, the protein of HMGB1 was also increased in DDP-resistant CC cells (Fig. 6c). In addition, correlation analysis results showed that mRNA HMGB1 expression was negatively correlated with miR-186 (R = − 0.7196, *P *< 0.0001), but positively correlated with NNT-AS1 (R = 0.6983, *P *< 0.0001) (Fig. 6d, e). Therefore, we hypothesized HMGB1 might involve in the NNT-AS1 medicated drug-resistant via miR-186. Subsequently, bioinformatics analysis was performed and HMGB1 was identified to be a candidate target gene of miR-186 (Fig. 6f). Afterwards, luciferase reporter assay results showed miR-186 mimic transfection reduced the luciferase activities of the HMGB1-WT reporter vector but not empty vector or HMGB1-MUT reporter vector in HeLa/DDP and SiHa/DDP cells (Fig. 6g, h). In the meanwhile, qRT-PCR and western blot results displayed HMGB1 expression, whether mRNA or protein, was inhibited by miR-186 mimic transfection, but was accelerated by NNT-AS1 transfection in HeLa/DDP and SiHa/DDP cells (Fig. 6i–l), indicating NNT-AS1 could regulate HMGB1 expression via modulating miR-186. Overall, NNT-AS1 could upregulate HMGB1 expression via targeting miR-186.

### Knockdown of NNT-AS1 antagonizes DDP resistance in DDP-resistant CC cells via regulating HMGB1

**Fig. 7:**
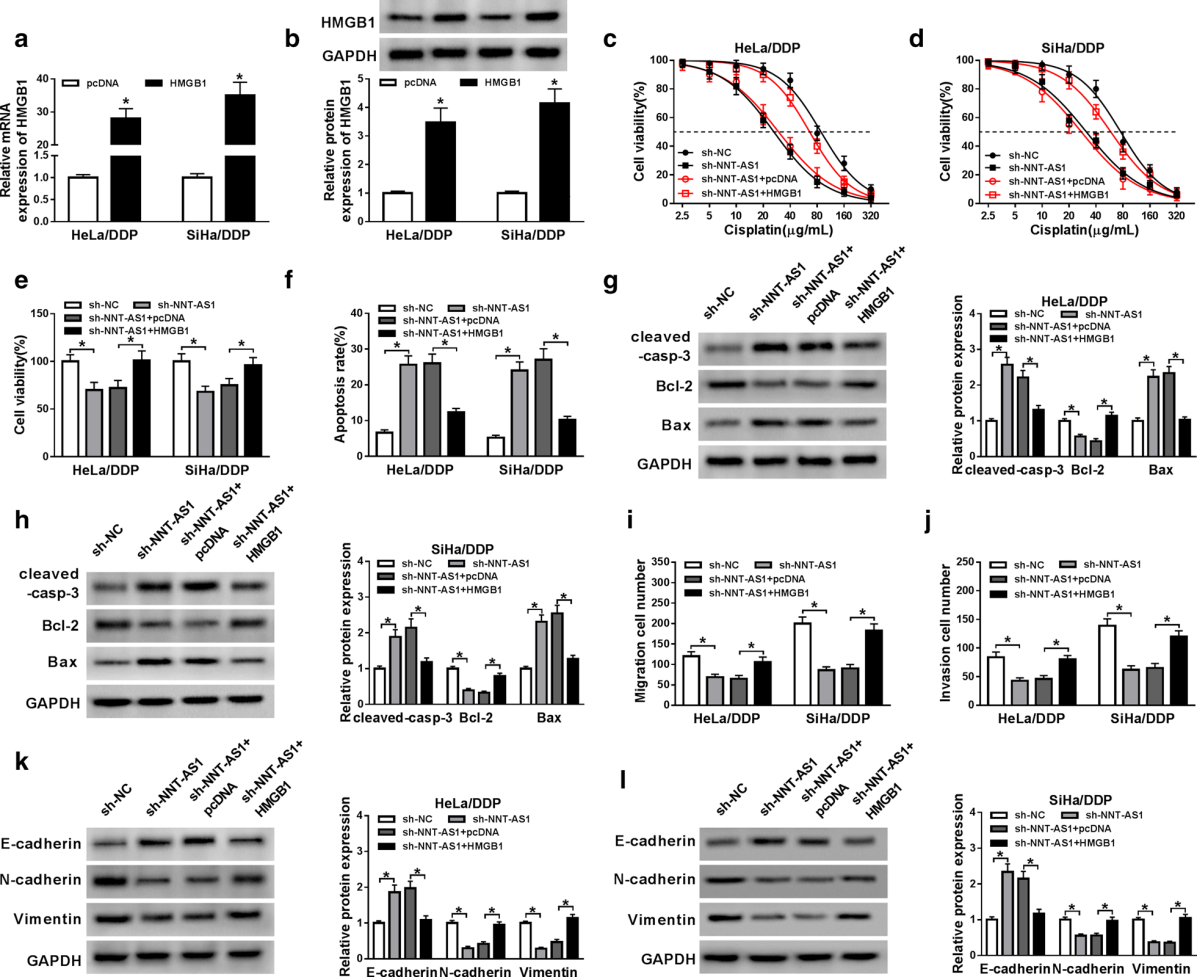
Knockdown of NNT-AS1 antagonizes DDP resistance in DDP-resistant CC cells via regulating HMGB1. **a**, **b** The mRNA and protein expression of HMGB1 in HeLa/DDP and SiHa/DDP cells transfected with sh-NC, sh- NNT-AS1, sh-NNT-AS1 + HMGB1 or sh-NNT-AS1 + pcDNA was measured using qRT-PCR or western blot, respectively. **c**–**e** Cell viability and proliferation of HeLa/DDP and SiHa/DDP cells were detected by MTT assay. **f** The apoptosis rate was examined by Flow Cytometry in HeLa/DDP and SiHa/DDP cells. **g**, **h** The protein expression of cleaved caspase-3, Bcl-2 and Bax in HeLa/DDP and SiHa/DDP cells was detected by western blot. **i**, **j** Transwell assay was used to analyze migration and invasion abilities of HeLa/DDP and SiHa/DDP cells. **k**, **l** The levels of E-cadherin N-cadherin and Vimentin were detected by western blot. *P < 0.05

To validate whether HMGB1 involved in the NNT-AS1-mediated cisplatin resistance, HeLa/DDP and SiHa/DDP cells were transfected with sh-NC, sh-NNT-AS1, sh-NNT-AS1 + HMGB1 or sh-NNT-AS1 + pcDNA. Then the transfection efficacy was verified by qRT-PCR and western blot with the results of increased HMGB1expression in cells transfected with HMGB1 (Fig. 7a, b). Immediately, the proliferation, apoptosis, migration, invasion and EMT abilities of DDP-resistant cells were investigated through restoration experiments. Actually, results indicated that HMGB1 transfection could attenuate NNT-AS1 deletion-mediated inhibition of proliferation, migration, invasion and EMT abilities as well as the promotion of apoptosis abilities in HeLa/DDP and SiHa/DDP cells (Fig. 7c–l). Thus, we demonstrated that knockdown of NNT-AS1 might antagonize cisplatin resistance in cisplatin-resistant CC cells by regulating HMGB1 expression. In addition, based on the above results, NNT-AS1 knockdown might improve DDP-sensitivity of CC via blocking HMGB1 expression by competitive interaction with miR-186.

### Knockdown of NNT-AS1 antagonizes DDP resistance in vivo

**Fig. 8:**
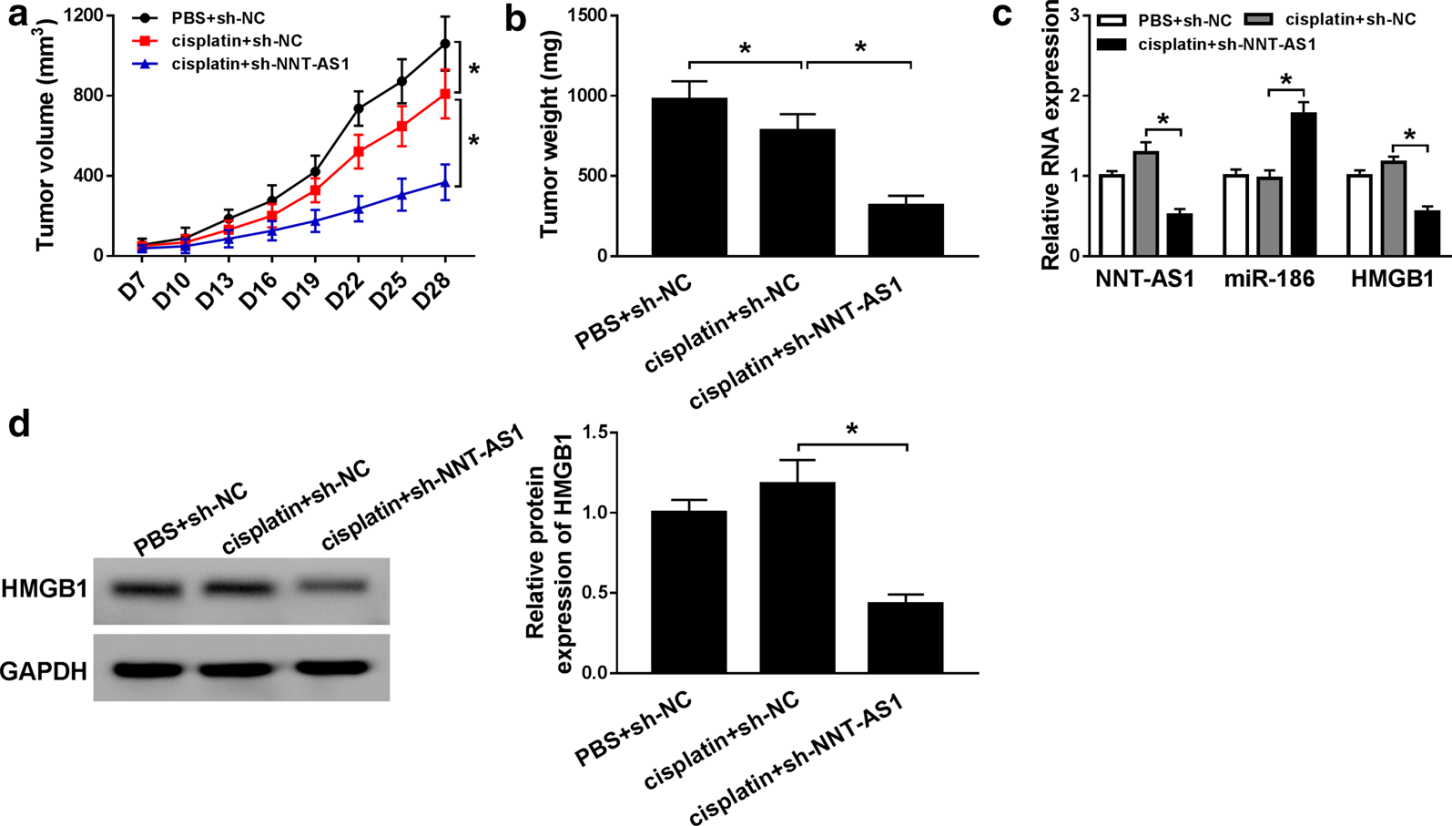
Regulatory effects of NNT-AS1 silence on tumor growth and miR-186 or HMGB1 expression in vivo. **a** Tumor volume was assessed every 3 days following DDP-treatment. **b** Tumor weights were measured in different groups. **c**, **d** The expression of NNT-AS1, miR-186 and HMGB1 in lumps was measured by qRT-PCR or western blot, respectively. *P < 0.05

A xenograft tumor mouse model was established to identify the impact of NNT-AS1 on DDP resistance in vivo. SiHa/DDP cells transfected with sh-NNT-AS1 or sh-NC were subcutaneously injected into BALB/c nude mice, following by the intraperitoneal injection of DDP or PBS. Then, we found NNT-AS1 knockdown significantly prevented tumor volume and weight (Fig. 7a, b). Subsequently, qRT-PCR analysis revealed that sh-NNT-AS1 transfection decreased NNT-AS1 expression in vivo, besides that, the expression of miR-186 was increased, while HMGB1 mRNA or protein expression was reduced in tumor tissues derived from sh-NNT-AS1-transfected CC cells (Fig. 8c, d). Thus, it was concluded that knockdown of NNT-AS1 might antagonize DDP resistance in vivo via regulating miR-186 and HMGB1 expression.

## Discussion

Since its discovery, DDP is considered as a pivotal drug in chemotherapy for cancers [25]. Nevertheless, it has been an impediment in using DDP for cancer treatment because of the chemoresistance in cancers [9]. Unsurprisingly, the treatment of CC is also plagued by drug resistance. Recently, numerous studies have identified the association between the dysregulation of lncRNAs and DDP resistance in cancers, including CC, and many lncRNAs has been investigated to be implicated in the DDP resistance. For instance, lncRNA UCA1 promotes DDP resistance in CC by involving in signaling pathways modulating cell apoptosis and proliferation [26]. LncRNA GAS5, acts as a tumor suppressor, can block DDP resistance in CC via miR-21 [27]. LncRNA ZFAS1 is upregulated in CC tissues, and its high expression indicates a poor prognosis and enhances DDP chemoresistance [2]. All the studies revealed that lncRNAs participate in the DDP resistance in CC. NNT-AS1, a newly detected lncRNA, has been identified to play important roles in tumor progression by affecting cell proliferation, metastasis, apoptosis and tumorigenesis in many cancers via targeting downstream signaling pathway, including CC [14, 28–30]. While the effects of NNT-AS1 on CC prognosis and DDP resistance remain unknown.

In the present study, we demonstrated that NNT-AS1 was highly expressed in CC tissues and cells, which was consistent with previous study. In the meanwhile, especially elevated of NNT-AS1 expression in DDP-resistant tumors and cell lines was also detected, suggesting the potential regulatory role of NNT-AS1 in DDP resistance of CC. The resistance towards DDP is multifaceted since it implicates multiple cellular pathways. Therefore, proliferation, migration, invasion and apoptosis abilities of DDP-resistant CC cells was evaluated and we found knockdown of NNT-AS1 could attenuate DDP resistance by inhibiting proliferation, metastasis and but inducing apoptosis in DDP-resistant CC cells. Additionally, the acquisition of EMT features has been investigated that contributes to the DDP-resistance in cancer cells including CC. EMT was revealed to play an important role in promoting chemoresistance [31, 32]. Thus, we also illustrated the expression levels of EMT related protein in CC DDP-resistant cells and an inhibition of N-cadherin and Vimentin expression but enhancement of E-cadherin expression induced by NNT-AS1 deletion in DDP cells, indicating NNT-AS1 stimulated EMT and induced DDP resistance in CC cells. Besides that, a murine xenograft model was established using stably transfected SiHa/DDP cells and the results showed that NNT-AS1 knockdown significantly prevented tumor volume and weight, suggesting NNT-AS1 deletion antagonized DDP resistance in vivo, which was consistent with the results in vitro. In consideration of the above-described biological behaviors of NNT-AS1, the relationship between NNT-AS1 and overall survival was evaluated and highly expressed NNT-AS1 was identified to predicate poor prognosis in CC patients.

It has been reported that lncRNA could act as competing endogenous RNA (ceRNA) of miRNAs to indirectly regulate gene expression implicated in various biological processes, including cancer [33]. MiR-186 was found to be a target of NNT-AS1 using bioinformatics prediction program and then NNT-AS1 directly binding to miR-186 was verified by luciferase reporter assay and RNA pull down assay. Subsequently, miR-186 expression was analyzed and miR-186 was demonstrated to be down-regulated in CC tissues and cell lines, especially in DDP-resistant tumors and cell lines. Immediately, a perfect negative correlation between miR-186 and NNT-AS1 expression in CC patients was identified. In all, all the results indicated that miR-186 might relate to NNT-AS1-mediated DDP-resistant.

MiR-186 has been investigated to act as a tumor suppressor to regulate proliferation, metastasis, apoptosis and EMT in CC cells [34, 35]. Nevertheless, the role of miR-186 in DDP resistance in CC remains unclear. Thus, to verify whether miR-186 related to NNT-AS1-mediated DDP-resistant, rescue experiments were performed and the data showed that miR-186 mimic transfection could restore NNT-AS1 deletion induced inhibition of DDP resistance in DDP-resistant CC cells. Therefore, NNT-AS1 improved DDP resistance in DDP-resistant CC cells by targeting miR-186.

An increasing number of findings suggests that aberrantly expressed miRNAs promote the development of drug resistance through interfering with the expression of target proteins which may be drug targets, drug transporters, or cell-cycle- and cell apoptosis-related components, causing cells with different degrees of resistant to chemotherapeutic drugs [36]. HMGB1 has been identified that may function as a DDP-resistant regulator in DDP resistance and inhibiting the cytoplasmic location of HMGB1 can reverse DDP resistance in human CC cells [37]. In this study, bioinformatics prediction program and luciferase reporter assay predicted and validated that HMGB1 was a target of miR-186. Moreover, expression analysis showed that NNT-AS1 could regulate HMGB1 expression via modulating miR-186 expression in vivo and vitro. Thus, we hypothesized HMGB1 might associated with NNT-AS1/miR-186 mediated DDP-resistant. Immediately, rescue experiments was performed and we found knockdown of NNT-AS1 could antagonize DDP resistance in DDP-resistant CC cells via regulating HMGB1. Therefore, NNT-AS1 knockdown might improve DDP-sensitivity of CC cells via blocking HMGB1 expression by competitive interaction with miR-186.

## Conclusion

We illustrated that NNT-AS1 was elevated in DDP-resistant tumor tissues and cell lines in CC, and highly expressed NNT-AS1 was associated with worse prognosis in CC patients. In addition, NNT-AS1 contributed to the DDP resistance in CC cells through NNT-AS1/miR-186/HMGB1 axis, indicating a novel therapeutic strategy to improve the efficacy of DDP in CC chemotherapy.

## Data Availability

The data sets used and/or analyzed during the current study are available from the corresponding author on reasonable request.

## Abbreviations

DDP: Cisplatin
CC: Cervical cancer
NNT-AS1: Nicotinamide nucleotide transhydrogenase-antisense RNA1
miR-186: MicroRNA-186
qRT-PCR: Quantitative real-time polymerase chain reaction
lncRNAs: Long non-coding RNAs
3′UTR: 3′-untranslated region
HMGB1: High mobility group box 1
EMT: Epithelial-mesenchymal transition
